# Turning Disaster into an Opportunity for Quality Improvement in Essential Intrapartum and Newborn Care Services in the Philippines: Pre- to Posttraining Assessments

**DOI:** 10.1155/2016/6264249

**Published:** 2016-06-15

**Authors:** M. S. Castillo, M. A. Corsino, A. P. Calibo, W. Zeck, D. S. Capili, L. C. Andrade, K. A. Reyes, R. C. Alfonso, M. B. Ponferrada, M. A. Silvestre

**Affiliations:** ^1^UNICEF Philippines, 1200 Makati, Philippines; ^2^Kalusugan ng Mag-Ina, Inc. (Health of Mother and Child), 1103 Quezon City, Philippines; ^3^Remedios T. Romualdez Medical Foundation (RTRMF), College of Medicine, 6500 Tacloban, Philippines; ^4^Department of Health Disease Prevention and Control Bureau, 1003 Manila, Philippines; ^5^Department of Obstetrics and Gynaecology, Medical University of Graz, 8010 Graz, Austria; ^6^Department of Health Regional Health Office 8 (Eastern Visayas), Palo, 6501 Leyte, Philippines; ^7^Alliance for Improving Health Outcomes Inc., 1104 Quezon City, Philippines

## Abstract

*Background.* On 8 November 2013, supertyphoon Haiyan made landfall in the Philippines, severely disrupting health service delivery. Reestablishment of essential services for birthing mothers and their newborns became high priority.* Methodology.* Following a baseline assessment, an Essential Intrapartum and Newborn Care (EINC) training package was implemented and posttraining assessments (1 and 3 months after training) were undertaken.* Results.* Baseline assessments (*n* = 56 facilities) revealed gaps in provider's skill and shortage of life-saving commodities. Facilities lacked newborn bags/masks (9%), towels (6%), and magnesium sulfate (39%). Service providers lacked skills in partograph use (54%), antenatal steroid (44%) use, and breastfeeding initiation (50%). At 3 months after training (*n* = 51 facilities), dramatic increases in correct partograph use (to 92%), antenatal steroid use (to 98%), breastfeeding initiation (to 86%), kangaroo mother care (to 94%), availability of magnesium sulfate (to 94%), and bag/masks (to 88%) were documented. Gaps persisted for skills in assisted vaginal delivery and removal of placental fragments.* Conclusion.* Health services were severely disrupted after supertyphoon Haiyan. Our study demonstrates that essential birthing services and quality improvements to strengthen local health systems can be restored in a timely manner even in immediate postdisaster settings.

## 1. Introduction 

Supertyphoon Haiyan made landfall in the Philippines on November 8, 2013, with most of the damages sustained in the central part of the country. 14.1 million people were affected, mostly in Eastern Visayas (Region 8), followed by Central Visayas (Region 7) and Western Visayas (Region 6) [1]. Immediate response efforts for vast areas ravaged by Haiyan were extremely difficult, due to breakdown of transportation and communication infrastructure, and consisted largely of provision of food and water, infant and child feeding, and triage and treatment for traumatic injuries and acute medical illnesses [2, 3]. Demand for health services surged as complete devastation of health infrastructure, logistics, and human resources rendered the local health system unable to respond. National and local government and interagency assessments immediately after the typhoon identified the reestablishment of health services for mothers and children, particularly primary and secondary care for obstetric emergencies, as a priority. The Emergency Health Cluster led by the Department of Health (DOH) (co-led by the World Health Organization, WHO) provided recommendations and supported appropriate humanitarian responses. Among the WHO endorsed interventions for saving the lives of mothers and children is the so-called Essential Intrapartum and Newborn Care (EINC), a package of cost-effective time-bound interventions [4]. In the Philippines, EINC (popularly known as “Unang Yakap” or the First Embrace) is implemented and mainstreamed in practice protocols or tools [5] following quality improvement principles [6]. The development of this standardized protocol was triggered by data from observational assessments in 51 government hospitals in the Philippines revealing that practices in the immediate newborn care period were undermining thermoregulation and breastfeeding initiation of newborns [7]. The importance of providing quality care for women during labor and delivery and implementation of safe practices for their newborns to ensure their best outcomes became paramount [8]. Prior to typhoon Haiyan, implementation of the EINC Protocol was being undertaken only in selected government hospitals and primary level facilities (rural health units and district hospitals). Inequity and system barriers to bring implementation to scale persisted despite national health policies being in place [9]. Neonatal mortality rate (NMR) reported in the National Demographic and Health Survey (NDHS 2013) [10] was 13 per 1000 live births nationally and 15, 18, and 10 per 1000 live births in Western, Central, and Eastern Visayas regions, respectively.

When a 7.2 magnitude earthquake jolted the province of Bohol in the Visayas region one month prior to typhoon Haiyan, WHO supported the rapid building of health worker capacity for maternal/newborn care in a postdisaster setting. EINC training packages were updated to incorporate new WHO guidelines on basic newborn resuscitation and postnatal care of mothers and newborns, including magnesium sulfate administration, to address gaps in care. After a pilot implementation in Bohol province, the training modules with the expanded content were endorsed by WHO for utilization in postdisaster areas where similar needs existed.

The aim of this intervention was to support the reestablishment of health care services for birthing mothers and their newborns in the areas affected by typhoon Haiyan guided by quality improvement principles. Specific objectives were to

1. conduct EINC training of trainers (TOT) and quality assurance (QA) workshops in target areas within Eastern, Central, and Western Visayas regions to address gaps identified in the baseline assessment
2. and undertake rapid assessments of EINC services using a standard tool at baseline and at 1 and 3 months after the training.

## 2. Materials and Methods

Based on a multistage vulnerability analysis, UNICEF and partners identified forty (40) priority municipalities along the path of typhoon Haiyan where the humanitarian response would be intensified during the period from February to September 2014.

This multistage vulnerability analysis entailed several stages: in the first stage, an initial list of 120 areas (cities and municipalities) was constituted based on preliminary data from the Philippines National Disaster Risk Reduction and Management Council (NDRRMC) and United Nations Office for the Coordination of Humanitarian Affairs (OCHA). Criteria used were (a) areas with 95% and above affected population, (b) areas given typhoon signal number 4, and (c) areas with highest potential for storm surge. Typhoon storm signal number 4 signifies a very intense typhoon with winds of more than 185 kph speed [11]. In the second stage, official data such as detailed population pre- and post-Haiyan, poverty incidence, and local data were incorporated. In the third stage, convergence of UNICEF interventions in the sectors of health, nutrition, education, child protection, and water, sanitation and hygiene (WASH) were analyzed to find the “best” model for prioritization of areas. In the fourth and final stage, the 40 priority municipalities were identified based on cumulative affected population that would yield optimal coverage in relation to UN strategic response plan targets developed with the Philippine government. These were in Eastern Visayas (Region 8): in the province of Leyte—Tacloban City, Palo, Tanauan, Dulag, Burauen, Dagami, La Paz, Julita, Tabontabon, Carigara, Jaro, San Miguel, Alangalang, Pastrana, Mayorga, and MacArthur; in the provinces of Eastern and Western Samar—Guiuan, Salcedo, Hernani, Mercedes, Balangiga, Giporlos, Lawaan, Quinapondan, Balangkayan, Borongan City, and Marabut. Those included in Western Visayas (Region 6) were in the province of Capiz—Pontevedra, Pilar, President Roxas, Maayon, Ivisan, Panay, Panitan, and Jamindan; in the province of Iloilo—Concepcion, Sara, and Estancia. In Central Visayas (Region 7), Bantayan and Daanbantayan municipalities in the province of Cebu were included. Technical experts from a local nongovernmental organization (NGO; Kalusugan ng Mag-Ina or “Health of Mother and Child”) were engaged to roll out the EINC implementation and quality improvement capacity building/training activities in priority municipalities from February to September 2014. In 2011, the same NGO provided the technical assistance in the pilot implementation of EINC in the Eastern Visayas Regional Medical Center in Leyte and the subsequent training of trainers and quality assurance workshop cascaded by the Region 8 Health Office for all Eastern Visayas provinces in 2012.

**Figure 1:**
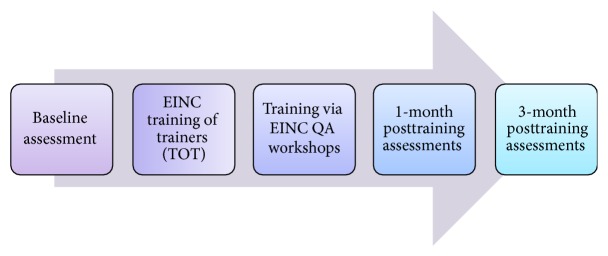
Flow of assessments and interventions.

The methodology to facilitate the capacity building of health workers in the forty (40) priority municipalities consisted of a training of trainers and cascade quality assurance (QA) workshops, with rapid assessment before the training and at one and three months posttraining (see Figure 1).

Using the “Newborn Services Rapid Health Facility Assessment” tool that was developed by the Interagency Newborn Indicators Technical Working Group as a template [12], rapid facility assessments were done at baseline (before training) and at one month and three months after training. Technical experts acting as external assessors used the identical tool to conduct pre- and posttraining assessments during facility visits. External assessors performed ocular surveys of the access and quality of the labor-delivery and postpartum environments, equipment, supplies, and patient pathways. Information was collected through interviews of health workers and mothers, supplemented by review of records. Whenever possible, deliveries were observed.

In the priority municipalities, primary level health facilities with the highest number of deliveries were intentionally selected. Based on usual patient traffic, the biggest district hospital or rural health unit (RHU) and its corresponding busiest lying-in clinic were selected, subject to the limited accessibility of facilities after typhoon Haiyan. In consideration of service delivery networks and referral flows within and across municipalities, assessments covered additional municipalities beyond the 40 priority areas.

The tool was used to assess facility-based delivery and newborn care service capacity in primary and referral level facilities. Gaps in newborn care health services were systematically identified using tracer indicators. Findings were utilized to describe capacity to address three main causes of newborn deaths: asphyxia, prematurity, and infection. Determinants were classified as (a) service availability, (b) equipment and supplies, and (c) service standards.

### 2.1. EINC Training of Trainers

Updated EINC training modules contained brief didactic sessions on basic topics: Essential Intrapartum and Newborn Care, breastfeeding support, kangaroo mother care, Infant and Young Child Feeding in Emergencies (IYCF-E), partograph use, and the recent WHO recommendations on basic newborn resuscitation, correct administration of magnesium sulfate, and postnatal care of mothers and newborns. Skills sessions consisted of “coaching sessions” with demonstration/return demonstrations using manikins and delivery kits. Workshops culminated with action planning sessions on effective mainstreaming of EINC in their practice. These planning sessions allowed the participants to voice out their concerns and their apprehensions and discuss among themselves ways to contextualize these challenges and address them.

Training of trainers (TOT) workshops were conducted in the three regions. Selection of trainees was strategic to include senior health care workers with aptitude to become trainers in their respective service delivery networks (roughly corresponding to geopolitical Interlocal Health Zones or ILHZs). Representation of involved ILHZs was ensured as much as possible to create a pool of trainers to cascade the training locally. Local trainers were further chosen from this pool based on their performance during the TOT. Participants included thirty (30) trainees each from Leyte, Eastern and Western Samar, Capiz, Iloilo, and Cebu.

### 2.2. Cascade EINC Quality Assurance Workshops

The technical experts supervised and mentored the new local trainers as they implemented their own cascade quality assurance (QA) workshops. In each of the areas, two QA workshops of 30 participants each were conducted, targeting a total of 300 capacitated skilled birth attendants. QA workshop modules for service providers were delivered over 6 half-day sessions. Pre- to postworkshop acquisition of knowledge was measured through 15 item multiple choice question written quizzes and acquisition of skills in the classroom assessments through performance checklists.

### 2.3. Statistical Analysis

Epi Info*™* 6 was used for data analysis. Categorical variables at baseline, 1-month and 3-month after training, presented as proportions (%), were compared using Fisher's exact and *χ* ^2^ tests with significance set at *p* < 0.05.

## 3. Results and Discussion

**Figure 2:**
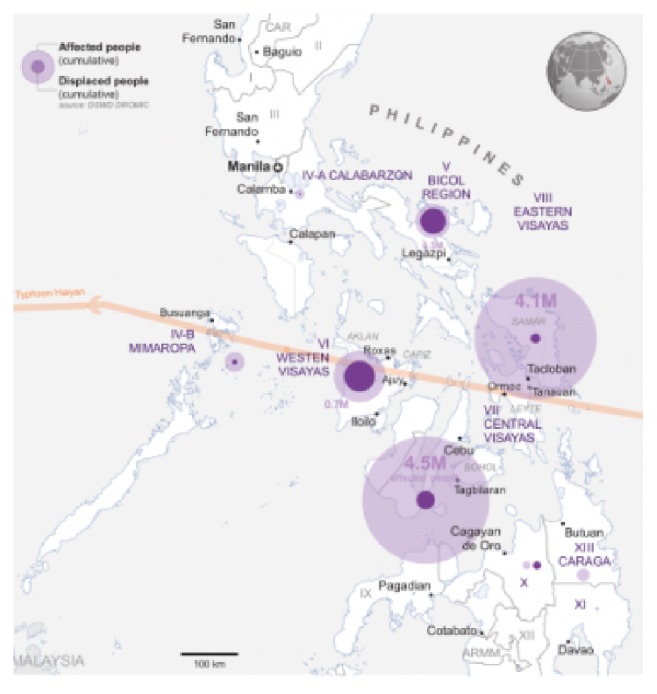
Path of typhoon Haiyan through central Philippines [15].

External experts supported the rehabilitation of EINC health services in 40 priority municipalities in Eastern, Central, and Western Visayas severely devastated by Haiyan (See Figure 2).

**Figure 3:**
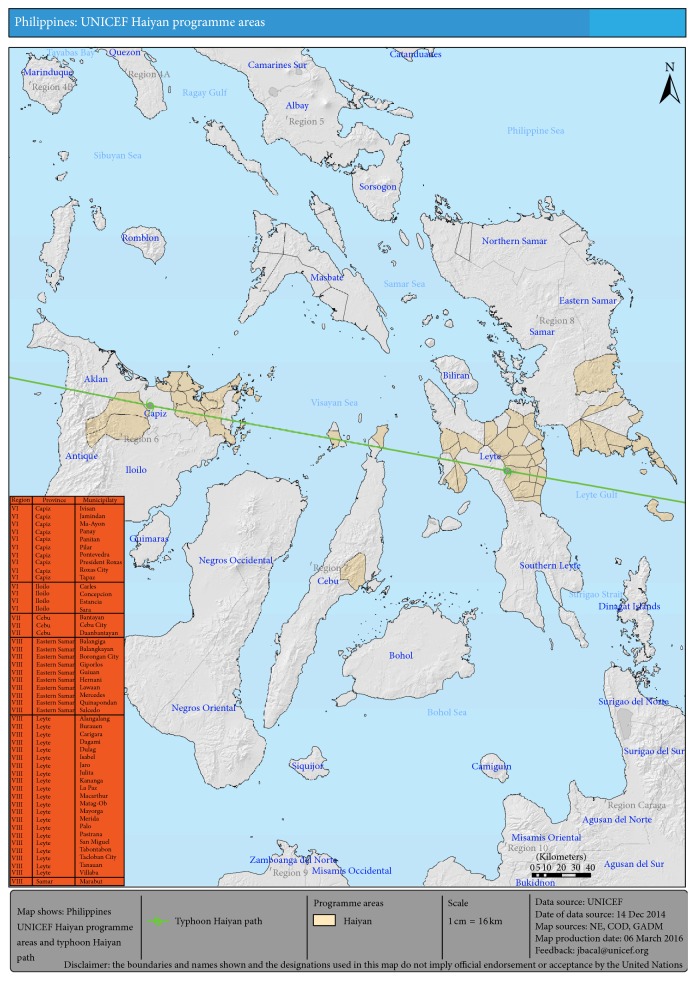
40 priority municipalities listed in orange box and shaded in map.

As part of the service delivery networks, additional areas adjacent to the priority municipalities were included, namely, four (4) municipalities and one city (Abuyog and Ormoc City in Leyte, Basey in Western Samar, and Barotac Viejo and Balasan in Iloilo). The grand total of 45 local government units (LGUs) thus included three (3) cities (in Leyte) and forty-two (42) municipalities (see Figure 3).

### 3.1. Baseline Assessments (Table 1)

Baseline rapid assessments were done at 16 weeks after landfall in Eastern Visayas (Leyte and Eastern and Western Samar), 20 weeks after landfall in Western Visayas (Iloilo and Capiz), and 22 weeks after landfall in Central Visayas (Cebu). A total of 56 health facilities were assessed at baseline. Assessments revealed significant disruption in obstetric and newborn health services in Eastern Visayas and much less significant disruption in Central and Western Visayas.

At baseline, health facilities assessed ranged from rural health units (RHU; 39), first level referral hospitals (15 community, district, and city hospitals), and, in addition, 2 provincial hospitals with birthing services. After typhoon Haiyan, the physical devastation in Eastern Visayas caused the most serious interruptions in EINC delivery. Immediate response services were provided primarily through contributions from international partners in tents and makeshift facilities. The lack of availability of 24/7 delivery services revealed the importance of improved referral mechanisms that could bring mothers experiencing complications during or after birth to a higher level facility, if needed. These findings differed from the assessment of Central and Western Visayas municipalities, where facilities mostly sustained partial damage with no interruption of birthing services.

Apart from damage to infrastructure, commodities were in serious shortage or absent with stock-outs of life-saving drugs (e.g., oxytocin, magnesium sulfate, and dexamethasone). Health human resources suffered serious setbacks not only due to substantial loss of lives among health care providers but also due to lack of training and technical capacity (in basic newborn resuscitation, care of the low birth weight, kangaroo mother care, breastfeeding, and Infant and Young Child Feeding in Emergencies (IYCF-E)). In facilities where health workers had participated in a previous 11-day Basic Emergency Obstetric and Newborn Care (BEmONC) training, low self-efficacy ratings especially in partograph use, magnesium sulfate administration, and newborn resuscitation rates were noted. Very few facilities experienced supervisory visits in the previous 6 months. None performed routine postnatal care services. Documentation of practices was weak. Finally, not all facilities conducted regular maternal and newborn death reviews.

### 3.2. EINC Training of Trainers (TOT)

Out of 150 candidate trainers who were invited, 112 (75%) attended the training. These were maternal and child health workers holding strategic positions (provincial level program managers, hospital specialists, municipal health officers, public health nurses, and midwife supervisors). A total of five (5) training of trainer (TOT) workshops of 3 days' duration each, were conducted, one each for the provinces of Leyte, Eastern and Western Samar, Iloilo, Capiz and Cebu.

### 3.3. Cascade EINC QA Workshops

As part of the training requirement, each TOT candidate trainer participated in at least one of a total of ten (10) cascade QA workshops for their colleagues, with supportive supervision and technical oversight from expert trainers. A total of 281 out of 300 targeted skilled birth attendants (93.7%) were further trained.

Thus, a total of 393 health workers completed EINC workshops over this period. 281 completed their participation in 3-day QA workshops and 112 attended the five TOT workshops. Consistent improvements in posttraining assessment quizzes and performance checklists for both the TOT and QA workshop participants were noted.

### 3.4. Posttraining Assessments (Table 1)

At 1 month and 3 months after training, field visits to 58 and 51 facilities, respectively, were carried out. The facilities that completed the 3 assessments were thirty-five (35) rural health units (RHUs), 15 primary level hospitals (13 district, 1 city, and 1 community hospitals) and one (1) provincial hospital. These field visits revealed that uptake of EINC knowledge and skills was translated to significant changes in daily practice. Posttraining assessment visits revealed good evidence of changes in the physical environments in the labor/delivery and postpartum areas and implementation of many of the EINC practices.

However, apart from the overall improvement, some posttraining gaps were identified: (1) not allowing a companion of choice, (2) nonadministration of birth doses of BCG vaccine, (3) unavailability of nevirapine therapy; (4) unavailability of protocols or guidelines for Integrated Management of Pregnancy and Childbirth (IMPAC), referral of sick newborns, Comprehensive Emergency Obstetric and Newborn Care (CEmONC), and preterm labor management; (5) absence of soap and hand disinfectants in postnatal areas.

### 3.5. Service Availability (Figure 4)

**Figure 4:**
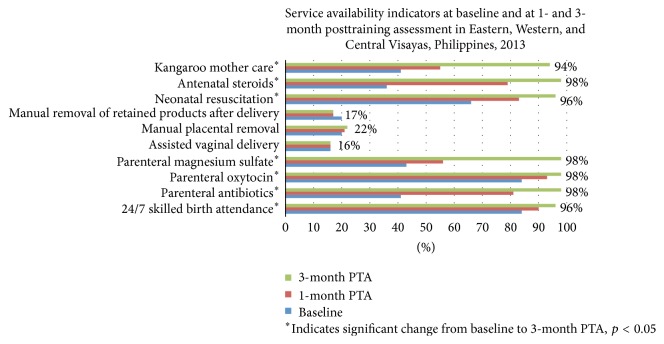
Proportion of facilities assessed providing various services at pretraining (baseline) and at 1 and 3 months posttraining assessment (PTA), Eastern, Western, and Central Visayas post-Haiyan.

**Figure 5 fig5:**
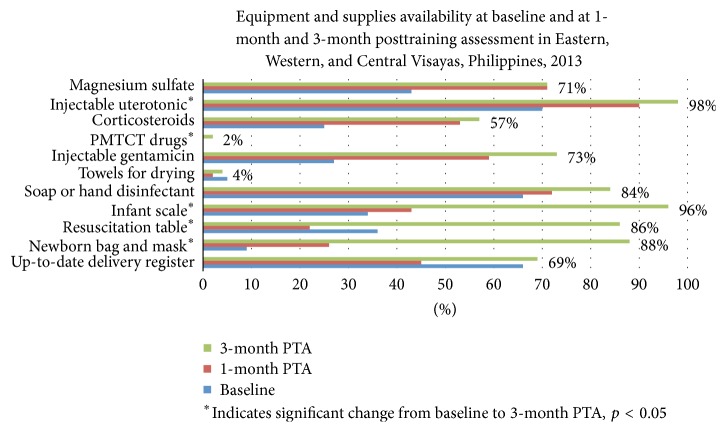
Proportion of facilities assessed to have various equipment and supplies at pretraining (baseline) and at 1 and 3 months posttraining assessment (PTA), Eastern, Western, and Central Visayas post-Haiyan.

**Figure 6:**
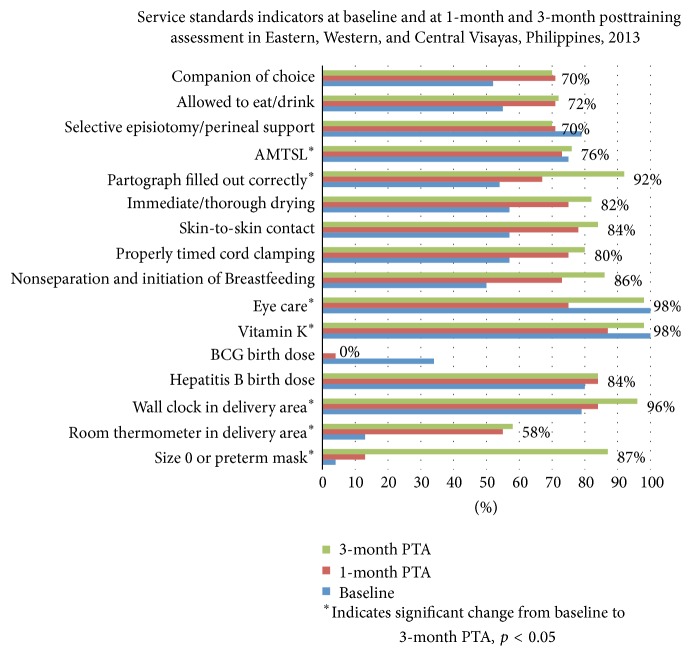
Proportion of facilities assessed to be implementing various service standards at pretraining (baseline) and at 1 and 3 months posttraining assessment (PTA), Eastern, Western, and Central Visayas post-Haiyan.

In Eastern Visayas, the worst hit region, baseline assessment revealed that 27 of 36 facilities were providing birthing services. At 1 month after training, 32 of 38 assessed birthing facilities were providing round-the-clock/daily (24/7) skilled birth attendance (SBA). At 3 months, 30 of 31 birthing facilities were providing 24/7 SBA. In Western Visayas, 16 of 16 facilities were able to sustain delivery services at baseline and 1 month assessments. One facility suspended its delivery services to undergo renovations at the 3-month assessment. All of the 15 other facilities were providing 24/7 SBA. In Central Visayas, all 4 facilities assessed were providing 24/7 SBA at baseline, 1-month, and 3-month assessments. Over the period from baseline assessment to 3 months after training, there were significant improvements in self-reported availability of services such as neonatal resuscitation, kangaroo mother care, administration of parenteral oxytocin, antibiotics, antenatal steroids, and magnesium sulfate. In contrast, persistent gaps remained for assisted vaginal delivery, manual placental removal, and manual removal of retained products after delivery. In Figures 4 [Fig fig5]–6, the proportion of facilities (%) at end line (3 months after training) is labeled.

### 3.6. Equipment and Supplies (Figure 5)

Over the period from baseline assessment to 3 months after training (20-week, 19-week, and 14-week periods for Eastern, Western, and Central Visayas, resp.), there were significant improvements in availability of life-saving drugs (i.e., oxytocin) and equipment (i.e., for resuscitation) most notably for Eastern and Central Visayas. For Western Visayas, some improvements were seen in the facilities but the limited availability of equipment constrained the appropriate delivery of services. Not all life-saving drugs were available in all facilities, especially antibiotics and steroids.

Though there was significant improvement, persistent gaps remained for antenatal steroids, injectable gentamicin, and magnesium sulfate supplies. Towels for drying were still being provided by families instead of by facilities. Nevirapine for prevention of mother-to-child transmission of HIV remained unavailable across all regions. Multidose BCG vaccines were available but considered “insufficient” by health workers. Many facilities had moved their weighing scales from their delivery areas to their maternity care areas for deferred weighing until after the first breastfeed, as recommended in EINC protocols. Protocols for IMPAC, sick newborn care/referral, preterm labor management, and CEmONC manuals remain unavailable.

### 3.7. Service Standards (Figure 6)

In addition to parameters included in the Newborn Care Services Rapid Assessment Tool, selected parameters relevant to the Philippines EINC Protocol and Basic Emergency Obstetric and Newborn Care (BEmONC) services standards were assessed through interviews of health service providers.

Significant improvements in service standards using tracer indicators were noted across all regions over the period from pretraining assessment to the 3 months posttraining assessment. Most notable were performance of the EINC “core steps” in immediate newborn care, proper WHO partograph use, and availability of size 0 face masks for resuscitation of preterms which improved from 0% to 100% at 3 months posttraining assessment.

Quality labor and delivery for mothers (companion of choice, allowing mothers to eat/drink, semiupright delivery bed, perineal support and restrictive episiotomy, and active management of the third stage of labor) and quality postpartum care (monitoring vital signs, uterine massage, and postpartum visit within first week and then within fourth to sixth weeks after discharge) were carried out. Facilities reported performance of the “core steps” of immediate newborn care (immediate and thorough drying, skin-to-skin contact, properly timed cord clamping, and nonseparation of newborn from mother for early breastfeeding), and routine newborn care (eye care, physical examination, and vitamin K administration). Postpartum women interviewed in the facilities verified the consistency of applying the EINC protocol.

A persistent gap in EINC standard practice was the birth dose of BCG vaccine. Ninety-six percent of facilities were not administering this birth dose as recommended by national guidelines citing that using the available multidose vials for only a few patients at each time constitutes “wastage.” This reflects the common practice of deferring the BCG vaccination to the first-postnatal visit in the Philippines. Number of room thermometers and wall clocks had increased in some facilities.

Unlike Western Visayas, the 4 facilities in Central Visayas had newborn resuscitator bags and masks available prior to the EINC intervention. With the intervention, however, health workers reported increased confidence in their newly acquired resuscitation skills. Wall clocks were present in delivery areas in all 4 facilities assessed from baseline to end line. Room thermometers, absent at baseline in all facilities, were available in all 4 facilities and end line.

Across the 3 regions, significant improvements in service standards were documented. In 2008, coverage of early skin-to-skin contact (SSC) was documented at 9.6% in delivery assessments in 51 government hospitals [7]. After government efforts at EINC scale-up, SSC coverage was at 64% by maternal report in 2013 [10]. In this study, SSC was at 84% in primary level facilities postdisaster by health worker report. Before Haiyan, barriers to EINC implementation, that is, absence of enabling physical and policy environments, noncompliance by specialists and other “experts” unfamiliar with the updated EINC practices, had been diminished but not eliminated. The devastation brought by Haiyan dismantled the systems that enabled EINC practice. Nevertheless, over a 3-month period, an intervention of training and posttraining assessments was able to restore and, for some EINC practices, even exceed pre-Haiyan coverage rates. The high coverage rates for EINC indicators at baseline suggests that previous scale-up efforts in EINC and other cross-cutting areas increased the potential for resilience. This complemented assistance offered by other humanitarian actors (e.g., infrastructure rehabilitation and provision of supplies and equipment) turning this into an opportunity to “build back better”. However, the contribution of pre-Haiyan capacity building efforts to this rapid restoration and further improvement of EINC services requires further evaluation. We postulate that if capacity building efforts have established enabling environments under normal circumstances, rehabilitation could be achieved in similarly brief time periods. Thus, capacity building for EINC may potentially be considered as an important disaster preparedness measure.

Aiming to improve the sustainability of this intervention, the external experts provided close supportive supervision during the cascade training sessions and the two posttraining assessments. Our experience reveals that both performance and environments have improved creating an enabling environment for EINC practices. There were still aspects that required improvement that could be reinforced by the local health officials during supervisory visits. However this can be a challenge in areas where regular supervision visits are rare due to difficulties in geographic access or security constraints.

Government support was provided through so-called Interlocal Health Zones (ILHZs), utilizing existing referral networks, often with rural health units (RHUs) and district hospitals as the focal point. Trainers were capacitated as ILHZ groups for subsequent roll-out through a skills transfer methodology. This approach had its limitation though, as trained health care providers from one ILHZ, in general, could not be tapped to train health care workers of another ILHZ.

In the aftermath of the disaster, several development and recovery activities resulted in competing schedules (e.g., water, sanitation, and hygiene (WASH), vaccine cold chain implementation, etc.) making it difficult to schedule training dates. Often, competing capacity building activities and the local rehabilitation efforts involved the same set of (limited) local manpower. Consequently, mobilizing local staffs to implement their trainers' knowledge in cascade QA workshops proved difficult. Another constraint posed the health human resource availability after the disaster since many health care workers were victims themselves.

The postdisaster setting likewise presented more challenges in conducting capacity building interventions. In some geographic areas communication and road infrastructure continued to be unusable making it difficult for health workers to attend trainings or gather local data. This postdisaster scenario constrained us to “pre- to postintervention assessment” and its inherent methodologic limitations.

This study was also limited by the fact that not all facilities who participated in baseline assessment were included in the posttraining assessment. Thus, this study could benefit from another evaluation after a longer interval posttraining to assess newborn outcome indicators, sustainability of improvements and further analysis on possible contributory effects of other sectoral interventions (e.g., WASH) and partner efforts in the areas. The contribution of predisaster capacity building to the results could not be studied.

## 4. Conclusions

This intervention filled the service gaps identified immediately after a large scale natural disaster and helped to reestablish and improve the delivery of health services in affected areas. Where baseline indices were worse because of the immediate effects of the typhoon (i.e., Eastern Visayas), the magnitude of improvement across most assessed parameters was more significant. However, there were other parameters where gaps persist despite intensive efforts and other methods and systemic interventions need to be explored address these deficiencies.

Our study demonstrates that quick important quality improvements can be made in a timely manner across a range of health facilities and with differing cadres of health workers, even in immediate postdisaster settings. Capacity building of health workers and strengthening of the local health system to deliver quality care before, during, and in the aftermath of a major disaster are among the preconditions for a resilient health system [13]. Building the evidence base linking these quality improvement efforts in EINC and basic newborn resuscitation with impact on newborn and maternal health outcomes postdisaster is imperative [14].

## Figures and Tables

**Table 1:** Number of facilities assessed at pretraining (baseline) and at 1 month and 3 months posttraining, by region and by province.

Region/province	Facility level		
	Rural health unit	First level hospital	Provincial hospital
*Eastern Visayas*			
*Leyte*			
Baseline	15	4	1
1 month	16	5	1
3 months	12	4	1
*Samar*			
Baseline	11	4	1
1 month	11	4	1
3 months	10	4	0

*Western Visayas*			
*Capiz*			
Baseline	3	3	0
1 month	3	3	0
3 months	3	3	0
*Iloilo*			
Baseline	8	2	0
1 month	8	2	0
3 months	8	2	0

*Central Visayas*			
*Cebu*			
Baseline	2	2	0
1 month	2	2	0
3 months	2	2	0

Progress in EINC implementation and quality improvement from baseline assessments through 1- and 3-month posttraining assessments.
